# Molecular Evidence of Genome Editing in a Mouse Model of Immunodeficiency

**DOI:** 10.1038/s41598-018-26439-9

**Published:** 2018-05-29

**Authors:** H. H. Abdul-Razak, C. J. Rocca, S. J. Howe, M. E. Alonso-Ferrero, J. Wang, R. Gabriel, C. C. Bartholomae, C. H. V. Gan, M. I. Garín, A. Roberts, M. P. Blundell, V. Prakash, F. J. Molina-Estevez, J. Pantoglou, G. Guenechea, M. C. Holmes, P. D. Gregory, C. Kinnon, C. von Kalle, M. Schmidt, J. A. Bueren, A. J. Thrasher, R. J. Yáñez-Muñoz

**Affiliations:** 1 AGCTlab.org, Centre for Gene and Cell Therapy, Centre for Biomedical Sciences, School of Biological Sciences, Royal Holloway, University of London, Egham, UK; 2 Infection, Immunity, Inflammation and Physiological Medicine Programme, Molecular and Cellular Immunology Section, UCL Great Ormond Street Institute of Child Health, University College London, London, UK; 3 Gene Transfer Technology Group, UCL Institute for Women’s Health, University College London, London, UK; 4 Sangamo Therapeutics, Inc., Richmond, California, USA; 5 Department of Translational Oncology, National Center for Tumor Diseases and German Cancer Research Center, Heidelberg, Germany; 6 Division of Hematopoietic Innovative Therapies, Centro de Investigaciones Energéticas, Medioambientales y Tecnológicas (CIEMAT)/Centro de Investigación Biomédica en Red de Enfermedades Raras (CIBERER-ISCIII)/Instituto de Investigación Sanitaria Fundación Jiménez Díaz (IIS-FJD, UAM), Madrid, Spain; 7 Department of Medical and Molecular Genetics, King’s College London, London, UK; 8 Great Ormond Street Hospital NHS Foundation Trust, London, UK

## Abstract

Genome editing is the introduction of directed modifications in the genome, a process boosted to therapeutic levels by designer nucleases. Building on the experience of *ex vivo* gene therapy for severe combined immunodeficiencies, it is likely that genome editing of haematopoietic stem/progenitor cells (HSPC) for correction of inherited blood diseases will be an early clinical application. We show molecular evidence of gene correction in a mouse model of primary immunodeficiency. *In vitro* experiments in DNA-dependent protein kinase catalytic subunit severe combined immunodeficiency (*Prkdc scid)* fibroblasts using designed zinc finger nucleases (ZFN) and a repair template demonstrated molecular and functional correction of the defect. Following transplantation of *ex vivo* gene-edited *Prkdc scid* HSPC, some of the recipient animals carried the expected genomic signature of ZFN-driven gene correction. In some primary and secondary transplant recipients we detected double-positive CD4/CD8 T-cells in thymus and single-positive T-cells in blood, but no other evidence of immune reconstitution. However, the leakiness of this model is a confounding factor for the interpretation of the possible T-cell reconstitution. Our results provide support for the feasibility of rescuing inherited blood disease by *ex vivo* genome editing followed by transplantation, and highlight some of the challenges.

## Introduction

Genome editing can repair defective genes, inactivate target genes and direct transgenes to safe harbours^1^. The targeted correction of mutations relies on homologous recombination (HR), a DNA repair pathway of insufficient efficiency for therapeutic approaches in primary cells^2^. Designer nucleases, which induce a double-strand break at the target locus and stimulate HR, can provide therapeutic-level genome editing^3^. *Ex vivo* genome editing of human haematopoietic stem/progenitor cells (HSPC) for correction of inherited blood diseases is feasible^4–7^, but clinical application should be preceded by demonstration of efficacy in animal models.

Primary immunodeficiencies (PIDs) are rare inherited disorders of the innate and acquired immune system. They result from more than 130 inherited mutations in genes required for production, differentiation or survival of specialised leukocytes like T or B lymphocytes, natural killer cells, neutrophils or antigen-presenting cells^8,9^. Patients regularly present with a higher vulnerability to opportunistic pathogens or infection with uncommon organisms and may also develop autoimmunity or autoinflammatory diseases and lymphoreticular malignancies^10^. Severe combined immunodeficiency (*scid*) disorders are fatal monogenic PIDs characterised by a severe decrease or lack of functional T-cells^11^. Typically, leukocytes originate from the multipotent HSPC in the bone marrow, so allogeneic haematopoietic stem cell transplantation (HSCT) is the preferred treatment. However; there are significant risks and some patients suffer from chronic complications, such as graft-*versus*-host disease^12^. Alternatively, promising gene therapy trials based on *ex vivo* retroviral-mediated gene addition prior to autologous HSCT have been performed in patients lacking a matched bone marrow donor^8^. The main obstacle facing this gene therapy approach is the possible risk of insertional mutagenesis leading to malignant outcomes^13^. Genome editing is a promising and more accurate approach, which may minimise the risk of unintended mutagenesis.

In this study the T- B- radiosensitive *scid* mouse^14^, a model of human DNA-dependent protein kinase catalytic subunit (DNA-PKcs; encoded by *PRKDC*) deficiency^15,16^, was used to provide proof-of-principle of phenotypic rescue by genome editing in the haematopoietic system. The *scid* mutation (T → A, Tyr-4046 → STOP) leads to an 83-amino acid C-terminal truncation which largely reduces protein stability of DNA-PKcs and kinase activity of DNA-PK^17,18^. This, in turn, causes failure of V(D)J recombination and hence T- B- immunodeficiency, and defective nonhomologous end-joining (NHEJ) resulting in radiation sensitivity^19^. This model was selected in the anticipation that *Prkdc*-corrected cells would have a selective survival advantage *in vivo* during the differentiation of T-cell progenitors. Mutant *Prkdc* has recently been corrected by genome editing in induced pluripotent stem cells, which have subsequently been differentiated to T-cells and characterised *in vitro*^20^.

We have used a ZFN and repair template to edit mutant *Prkdc* in mouse *scid* fibroblasts, and show evidence of correction at the molecular and functional levels. We then gene-edited *Prkdc* in *scid* HSPC and provide molecular evidence of correction. Upon transplantation to *scid* animals, gene-edited HSPC led to detectable levels of gene correction in several tissues of some of the recipients. In some cases we also observed double-positive CD4/CD8 T-cells in thymus, and single-positive T-cells in blood after primary and secondary transplants, but the evidence of cellular reconstitution is not entirely conclusive due to the leakiness of this model. We conclude that *Prkdc* gene-editing has been achieved in both *scid* fibroblasts and HSPC, and that the latter are able to mediate partial *in vivo* reconstitution, which can be detected at the molecular level and might lead to increased levels of T-cells in some of the recipients.

## Results

### *Prkdc* gene editing in *scid* fibroblasts

**Figure 1:**
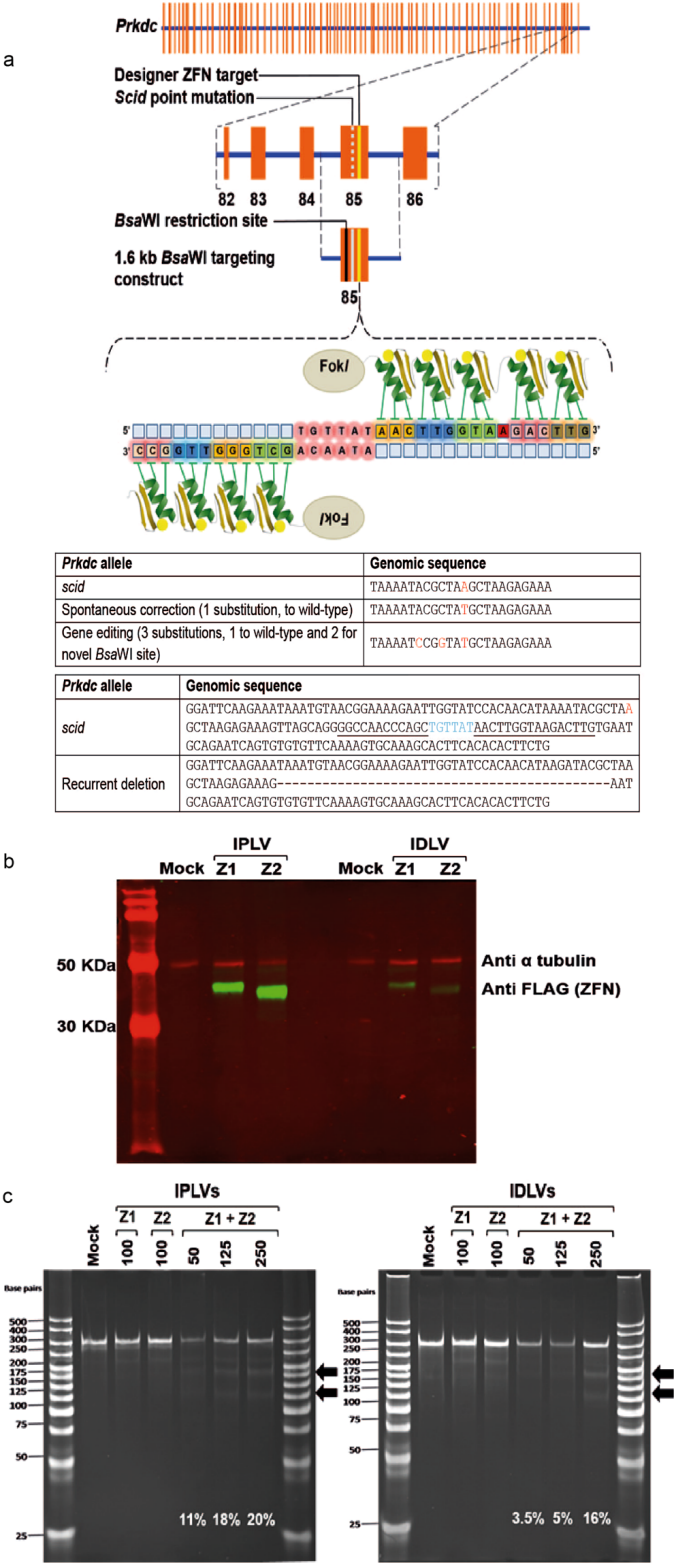
*Prkdc* gene editing strategy and outcomes, ZFN design and gene expression, and cutting activity in *scid* fibroblasts. (**a**) Schematic of *Prkdc*, including location of *scid* and ZFN target sites, structure of ZFN and sequences of various *Prkdc* alleles around *scid* site. (**b**) Production of ZFN monomers from SFFV-driven IPLV and IDLV (qPCR MOI 100). Proteins were extracted 3 d after transduction. The blotting revealed FLAG-tagged ZFN monomers (42 and 38.5 kDa for ZFN1 & ZFN2, respectively), with α-tubulin used as a loading control (49 kDa). (**c**) *Cel*-I analysis of *Prkdc* cutting by ZFN. *Scid* fibroblasts were transduced with IPLV- and IDLV-ZFN at the indicated MOI and genomic DNA was extracted 3 d later. The *Cel*-I-digested PCR products were separated on a polyacrylamide gel and diagnostic bands (black arrows) were used to calculate the % of indels induced by the various ZFN treatments and indicated on the gel image.

To edit *Prkdc*, we designed ZFNs to induce a DNA double stranded break (DSB) at this locus. We produced a ZFN heterodimer that recognizes a sequence in exon 85 of the murine *Prkdc* gene, 32 nucleotides downstream from the *scid* point mutation (Fig. 1a and Supplementary Figure 1). The ZFN monomers, carrying triple FLAG epitopes to allow facile detection by Western blotting, were cloned into lentiviral (LV) transfer plasmids (Supplementary Figure 2). Integration-Proficient Lentiviral Vectors (IPLVs) and Integration-Deficient Lentiviral Vectors (IDLVs) were produced and the expression of the ZFN monomers in *Tert*-immortalised *scid* fibroblasts was confirmed by Western blotting following LV transduction (Fig. 1b). Fibroblasts were immortalised for the purpose of obtaining clones for downstream analysis, as preliminary experiments had shown an inability of the primary cells to grow as clones. As expected from transductions of dividing cells^21^, this revealed strong expression from ZFN IPLVs and lower levels from ZFN IDLVs. To test the functionality of the ZFN at the target locus we used a mismatch-sensitive endonuclease assay, the Surveyor-nuclease (*Cel*-I) assay^22^. It should be noted that this assay underestimates the actual cutting frequency as most of the DSBs are repaired error-free^23^. With both IPLVs and IDLVs we observed dose-dependent increases in ZFN cutting activity, reaching 20% and 16%, respectively (Fig. 1c). Deep sequencing of the IPLV-treated sample at qPCR multiplicity of infection (MOI) 125 showed that 18% of the sequence reads carried small insertions and deletions at the site of ZFN cleavage (Table 1), corroborating the result of the *Cel*-I assay. As *Prkdc* is an autosomal gene, the percentages of indels or correction at the molecular level given throughout this paper would translate into approximately double the percentages of cells, if the vast majority of cells were gene modified at only a single allele. In practice, it has been shown previously that ~50% of the cells may be biallelically modified^3^.

**Table 1:** On-target and off-target cutting analysis of *Prkdc* ZFN in *scid* fibroblasts (transduced with IPLV-SFFV-ZFN, qPCR MOI 125) and HSPC (transduced with IPLV-SFFV-ZFN, qPCR MOI 2,500).

Site	Sample	%Indels mock	%Indels ZFN	%Differential (ZFN-mock)
On-target	Fibroblasts	0.05	18.02	17.97
Off-target 1	Fibroblasts	0.66	0.73	0.07
Off-target 2	Fibroblasts	0.10	0.21	0.11
Off-target 3	Fibroblasts	0.02	0.11	0.09
Off-target 4	Fibroblasts	0.11	0.10	−0.01
Off-target 5	Fibroblasts	0.06	0.14	0.08
Off-target 6	Fibroblasts	0.76	0.98	0.22
Off-target 7	Fibroblasts	0.00	0.01	0.01
Off-target 8	Fibroblasts	0.52	1.03	0.51
Off-target 9	Fibroblasts	0.01	1.35	1.34
On-target	HSPCs	0.08	0.60	0.52
Off-target 1	HSPCs	0.08	0.53	0.45
Off-target 2	HSPCs	0.08	0.16	0.08
Off-target 3	HSPCs	0.00	0.00	0.00
Off-target 4	HSPCs	0.02	0.03	0.01
Off-target 5	HSPCs	0.04	0.03	−0.01
Off-target 6	HSPCs	0.69	0.70	0.01
Off-target 7	HSPCs	0.01	ND	ND
Off-target 8	HSPCs	0.29	0.18	−0.11
Off-target 9	HSPCs	0.00	ND	ND

**Figure 2:**
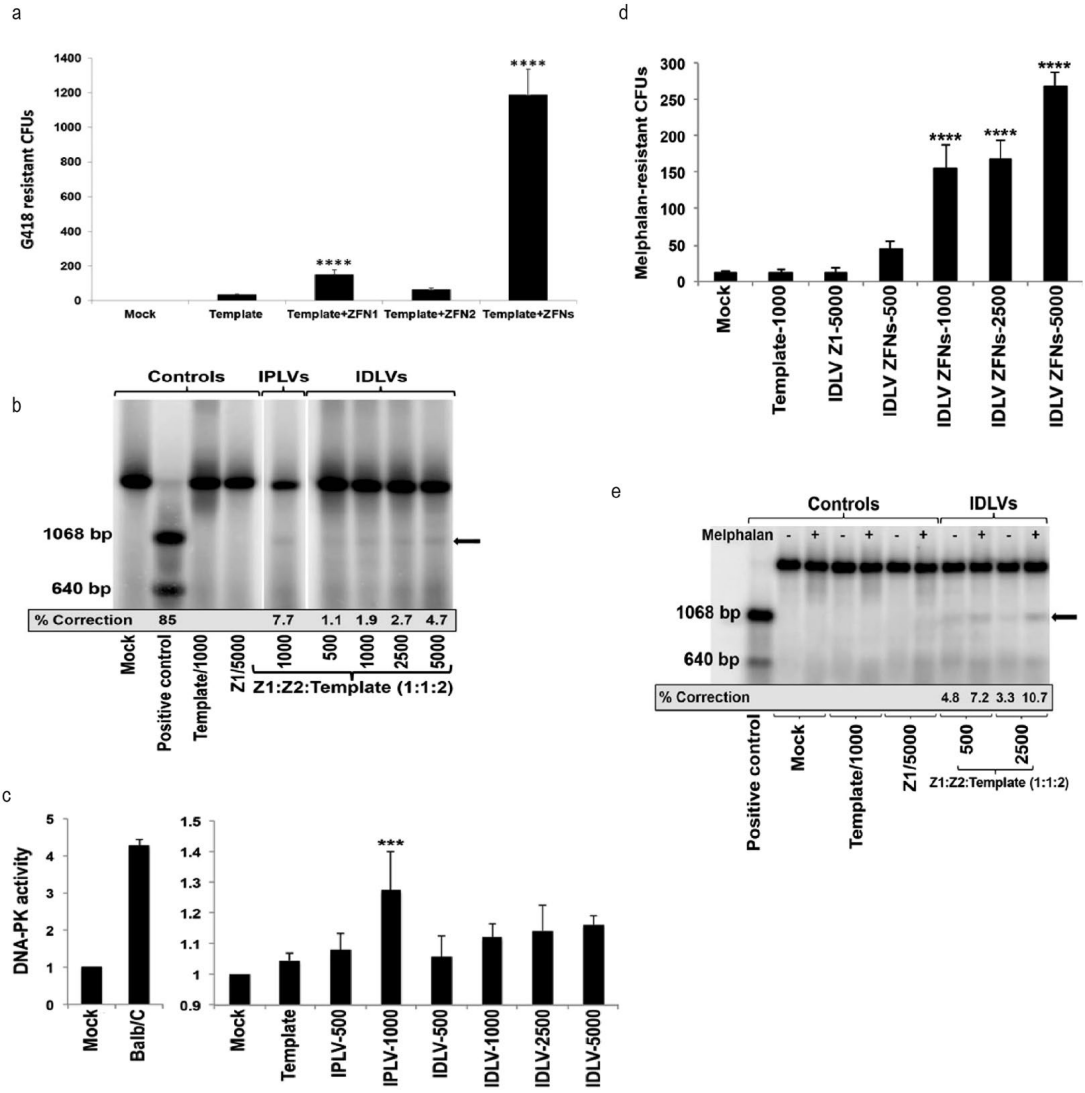
Phenotypic and molecular analyses of *Prkdc* gene editing in *scid* fibroblasts. (**a**) Gene editing in *scid* fibroblasts by plasmid transfection. *Prkdc*-*neo* template plasmid and ZFN monomer plasmids were used. All samples but the mock received repair template. G418-resistant CFUs were stained with crystal violet and counted. Statistical significance was evaluated using one-way ANOVA with Dunnett’s post hoc, comparing against template-only sample; ****p < 0.0001. (**b**) *Prkdc* gene editing in *scid* fibroblasts by transduction. Cells were transduced with IPLV-ZFN/IDLV-template or IDLV-ZFN/template at the indicated qPCR MOI and genomic DNA was extracted 10 d post-transduction. *Scid* locus was PCR-amplified with primers external to template, and ZFN-mediated gene correction was quantified from the diagnostic *Bsa*WI band (arrow) and shown as %*Prkdc* correction. All samples shown were run in the same gel, from which irrelevant lanes have been cropped at places shown as thin white strips. The uncropped gel is shown in Supplementary Figure 6. (**c**) Restoration of DNA-PK activity after ZFN-mediated gene correction. *Scid* fibroblasts were transduced as above with IPLV or IDLV ZFN/template at the indicated MOI. The DNA-PK activity assay was performed using nuclear proteins extracted 10 d post transduction. Specific DNA-PK enzyme activity is plotted as a ratio between sample (gene edited cells) and mock (*scid* fibroblasts). Statistical significance was evaluated using a one-way ANOVA with Dunnett’s post hoc, comparing against mock sample; ***p < 0.0005. (**d**) Enhanced resistance of *Prkdc* gene-edited cells to DNA damage. *Scid* fibroblasts transduced with IDLV-ZFN/template at the indicated MOI were exposed to 10 μM melphalan for 1 h weekly for 3 weeks, then colonies were stained with crystal violet and CFU scored. Statistical significance was evaluated using a one-way ANOVA with Dunnett’s post hoc, comparing against mock sample; ****p < 0.00005. (**e**) Melphalan enrichment correlates with increase in molecular signature of *Prkdc* gene editing. *Scid* fibroblasts transduced with IDLV-ZFN/template at the indicated MOI were maintained in culture with or without weekly 10 μM melphalan treatment. Genomic DNA was extracted 3 weeks post-transduction and subjected to *Bsa*WI digestion. The %*Prkdc* gene correction calculated from the intensity of the diagnostic band (arrow) is indicated on the gel.

A plasmid-based gene targeting experiment was performed to assess the frequency of ZFN-mediated homology-dependent repair (HDR) at the *Prkdc* locus. The *Prkdc* ZFN plasmids and a recombination template, carrying a 7.5 kb *Prkdc* genomic DNA including exons 82–86 and a neomycin phosphotransferase (*neo*) gene, were transfected separately or in combination into *Tert*-immortalised *scid* fibroblasts. *Neo* confers resistance to G418 and upon HDR will be targeted for insertion into intron 85 (Supplementary Figure 3). Random integration of the recombination template is also expected to produce a background of G418-resistant colonies. A significant ~30-fold increase in the number of G418-resistant colonies, to ~1.2% of total cells, was observed in the presence of both ZFN and template together compared to template alone (p < 0.0001; Fig. 2a). An inside-out PCR screening of 36 G418-resistant clones from the ZFN + template groups showed 20 of them to be *Prkdc*-targeted by HDR (Supplementary Figure 3), a percentage that is only achievable through either promotor-trap^2,24,25^ or designer nuclease-mediated strategies^3^. When SFFV or CMV-driven IPLV-ZFN and the plasmid template were used, the frequencies of G418-resistant colonies were approximately 50% of those obtained in the all-plasmid paradigm (not shown). This lower frequency could be related to sub-optimal experiments combining transduction/transfection, or increased cytotoxicity from IPLV-ZFN compared to plasmid-ZFN.

We then analysed gene correction at *Prkdc* by combined delivery of the ZFNs (via IPLV or IDLV) with a 5-fold shorter (1.6 kb, Fig. 1a) repair template suitable for *ex vivo* IDLV delivery strategies. In this template, a silent diagnostic *Bsa*WI restriction site has been introduced *via* site-directed mutagenesis two nucleotides upstream from the *scid* site (Fig. 1a and Supplementary Figure 1). Upon HDR-mediated gene editing, the *Bsa*WI site is incorporated into the target locus alongside the correct nucleotide at the *scid* site (Supplementary Figure 4) and can be subsequently used as diagnostic for gene editing^26^. For experiments with IPLV-ZFN the template for gene correction (the “donor” construct) was supplied on a separate IDLV; for experiments with IDLV-ZFN, the donor construct was present in both IDLVs carrying the nuclease monomer genes. The ZFN-mediated frequency of targeted gene correction reached 7.7% with IPLV delivery of the ZFN, and 4.7% with IDLVs (Fig. 2b). Deep sequencing of the IDLV sample at qPCR MOI 1,000 confirmed the presence of 1.53% corrected sequences including the diagnostic *Bsa*WI site and the wild-type nucleotide at the *scid* site, both of which result from ZFN-driven targeted correction.

Next, we used these pools of fibroblasts to determine whether ZFN-mediated gene correction of *Prkdc* led to restoration of DNA-PK activity, using a previously described assay^27^. Our results showed that DNA-PK activity in gene-corrected fibroblasts increased in a vector dose-dependent manner, reaching statistical significance for the IPLV at qPCR MOI 1,000 (Fig. 2c). *Prkdc* gene correction also led to increased resistance to melphalan-induced DNA interstrand cross-linking damage, the repair of which is dependent on an active DNA-PK complex^28^. We used 10 μM melphalan, which reduces the viability of *scid* fibroblasts by 80% and that of wild-type cells by 40% (Supplementary Figure 5). We observed a significant vector dose-dependent increase in the number of colony forming units (CFU) produced in the presence of melphalan by IDLV-gene edited *scid* fibroblasts (Fig. 2d). The increase in CFU was mirrored by an elevated level of the *Bsa*WI gel band diagnostic for gene correction, enriched up to 3.7-fold (from 3.3% to 10.7% *Prkdc* modification; Fig. 2e). No CFU were obtained from IPLV-ZFN-treated fibroblasts, suggestive of cytotoxicity associated with constitutive expression of the *Prkdc* ZFNs in fibroblasts.

### *Prkdc* gene editing in *scid* HSPC and transplantation

**Figure 3:**
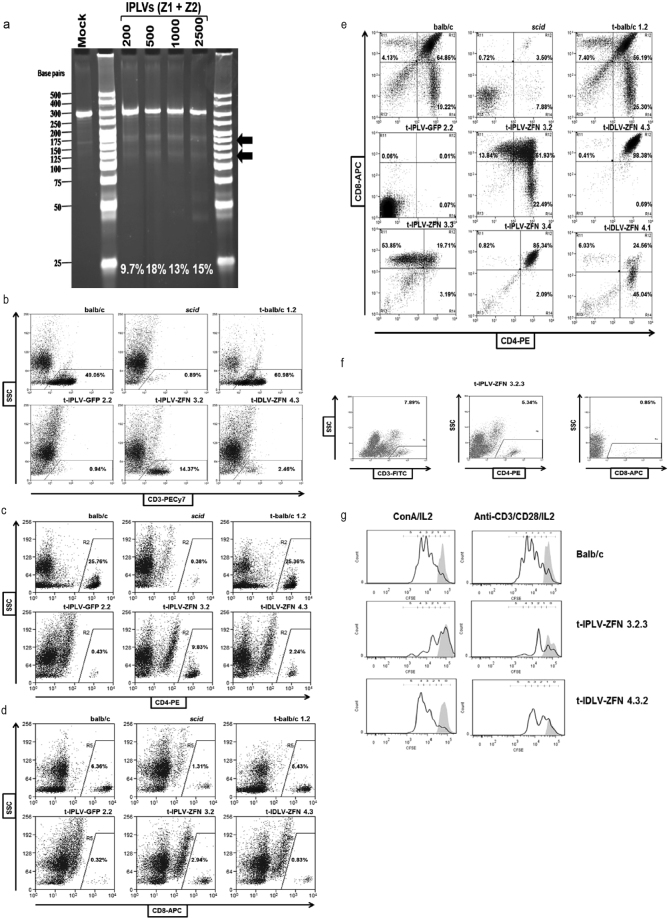
ZFN activity in HSPC and rescue of *scid* mouse T-cell deficiency. (**a**) *Cel*-I analysis of ZFN-mediated *Prkdc* cutting. *Scid* HSPC were transduced with IPLV-ZFN at the indicated MOI and genomic DNA was extracted 3 d later. Diagnostic *Cel*-I digestion products are indicated with black arrows and the % of indels induced by ZFN treatments shown on the gel image. (**b–d**) T-cell populations in PBMC from primary transplant recipients, 27 weeks post-transplantation. Primary transplantation of irradiated female *scid* mice was done with gene-edited male *scid* HSPC, while secondary transplantation of female *scid* mice was with whole bone marrow (BM) cells. Flow cytometry analyses of (**b**) CD3, (**c**) CD4 and (**d**) CD8 T-cell populations from wild-type balb/c and *scid* mice, and balb/c, *scid*-IPLV-eGFP, *scid*-IPLV-ZFN/IDLV-template and *scid*-IDLV-ZFN/template transplant recipients are shown. (**e**) Thymic T-cell precursors in primary transplant recipients, 27–32 weeks post-transplantation. 50% of gene-edited transplant recipients were responders with double-positive CD4 CD8 cells. (**f**) T-cell populations in PBMC of secondary *scid*-IPLV-ZFN/IDLV-template transplant recipient, 35 weeks post-transplantation. (**g**) Proliferative response of purified spleen CD3 T-cells at 35 weeks post-secondary transplantation. CFSE-stained T-cells were cultured in the presence (black line) or absence (grey overlay) of concanavalin A and IL-2 or anti-CD3/CD28 and IL-2. After 72 h, cell expansion of the T-cell population was assessed by CFSE dilution measured by flow cytometry.

To attempt *Prkdc* gene editing in HSPC we used lin^−^ *scid* bone marrow cells^29^. Using IPLV-delivered ZFN we detected up to 18% target gene cutting according to a *Cel*-I assay (Fig. 3a). *Prkdc* gene correction determined by deep sequencing ranged 4–17%. In contrast, when using IDLV, deep sequencing showed *Prkdc* indels with SFFV-ZFN peaking at ~0.4%, corroborating the known weakness of the SFFV promoter when used in the context of an IDLV vector to deliver transgenes^21^. Given the poor efficiency obtained with the SFFV promoter in the IDLV configuration, we performed a search for an optimised non-integrating delivery method including the use of a variety of promoters in IDLVs, and a number of adenoviral vectors. Optimal *eGFP* expression in HSPC (considering both transduction efficiency and mean fluorescence intensity) was obtained with IDLVs driven by a CMV promoter. In preliminary experiments, IDLV-mediated *Prkdc* gene correction in HSPC reached 2.1% of sequences bearing both the diagnostic *Bsa*WI site and the wild-type nucleotide at the *scid* site as detected by deep sequencing (data not shown).

Having determined that *Prkdc* gene-corrected HSPC could be obtained, we set up an experimental schedule (Supplementary Table 1) including *ex vivo Prkdc* gene editing in *scid* HSPC, primary transplantation into sub-lethally irradiated *scid* mice, and secondary transplantation of whole bone marrow into similarly irradiated *scid* mice, with periodic phenotypic analyses. HSPC were transduced with IPLV-SFFV-ZFN or IDLV-CMV-ZFN, albeit as shown above IDLV delivery of the ZFN monomers was clearly suboptimal in HSPC; the repair template was always provided in an IDLV as described above for fibroblasts. Control groups included animals receiving (i) a wild-type transplant of lin- HSPC, and (ii) lin- *scid* HSPC transduced with IPLV-eGFP. For molecular analyses, blood samples were collected at 10, 17 and 27 weeks post-transplantation from all primary transplant recipients, and at 9, 20 and 35 weeks from secondary transplant recipients. Peripheral blood mononuclear cells (PBMC) were analysed by flow cytometry to quantitate T-cells (CD3, CD4 and CD8), B-cells (B220) and myeloid (CD11b) cells. Table 2 summarises engraftment, vector copy number (VCN), phenotypical analyses and deep-sequencing data for *Prkdc* gene correction. As expected from animals receiving *scid* HSPC, the myeloid cells were considerably increased, as the recipients essentially did not develop B-cells and had varying levels of T-cells. VCNs were negligible in animals receiving transplants from IDLV-only treated cells, as these non-replicating vectors will dilute progressively with the cell population expansion.

**Table 2:** Engraftment, vector copy number, cytometry, gene editing status and deep sequencing results from transplant.

Observations		Bone marrow qPCR		Cytometry			*Prkdc* gene editing assessed by deep sequencing (% corrected sequences in genomic DNA)					
		Engraftment	VCN	Blood CD3+	Thymus CD4+CD8+	Spleen CD3+ proliferation	Status	Blood	Thymus	Spleen	Spleen CD8+	Spleen CD3+
**Primary Transplant**												
**Balb/c**												
1.2	secondary transplant donor	93.1%	NA									0.00%
**scid IPLV-eGFP**												
2.2	secondary transplant donor	100.0%	0.80									
**scid-IPLV-ZFN**												
3.2	secondary transplant donor	95.5%	0.48	+	+		+	5.42%, 5.80%	13.73%, 11.81%, 0.39%	0.45%	31.70%, 22.18%	
3.3	splenomegaly	27.2%	0.14	−	+		+		0.10%	0.01%		
3.4		47.9%	1.12	−	+		−		0.00%	0.00%		
**scid-IDLV-ZFN**												
4.1		89.0%	0.02	−	+		+		0.00%	0.02%, 0.07%		
4.2	splenomegaly			−	−		−		0.00%	0.00%		
4.3	secondary transplant donor	62.4%	0.01	+	+		+	0.01%	0.00%	0.03%	0.34%	
4.4				−	−		−		0.00%	0.00%		
4.5				−	−		+		0.00%	0.28%		
**Secondary Transplant**												
**Balb/c**												
1.2.1	donor: 1.2	64.6%	0.57									
**scid IPLV-eGFP**												
2.2.1	donor: 2.2	25.1%	0.14									
2.2.2.	donor: 2.2; leukemia	57.2%	0.20									
**scid-IPLV-ZFN**												
3.2.1	donor: 3.2	30.0%	0.64	+			+			2.70%		
3.2.2	donor: 3.2	77.3%	0.69	+	+		+		1.58%	0.93%		
3.2.3	donor: 3.2	35.9%	0.50	+		+	+	3.41%, 7.44%		8.77%		24.58%
**scid-IDLV-ZFN**												
4.3.1	donor: 4.3	4.2%	0.01	−/+	−/+	+	+	4.92%	0.00%	0.00%		0.00%
4.3.2	donor: 4.3	45.8%	0.01	−/+		+	+	0.01%		0.00%		0.00%
4.3.3	donor: 4.3; thymomegaly	5.5%	0.01	+	+		−	0.00%	0.00%	0.00%		

For Deep sequencing data, when several values are provided for a particular tissue they indicate the percentages obtained from separate samplings. Thymus and spleen, whole tissue genomic DNA was used as PCR template.

One primary recipient from each ZFN + template HSPC group, IPLV and IDLV, showed increasing levels of CD3 T-cells over time, reaching 14.37 and 2.46% of PBMC respectively (Fig. 3b and Table 2). Similar results were obtained with CD4 and CD8 T-cell stainings (Fig. 3c,d and Table 2). When we analysed the thymus of the primary transplant recipients, fifty percent of animals from the ZFN + template groups (3/5 from the integrating group and 2/5 from IDLV group) showed double-positive CD4/CD8 T-cells (Fig. 3e and Table 2). Some of these CD4/CD8 patterns were somewhat abnormal, with very high levels of double-positive cells. Spleen and bone marrow were also analysed at culling, with unremarkable results.

Unfractionated bone marrow cells from the best primary responders (mice presenting with the highest levels of CD3 and CD4/8) in groups treated with ZFN + template combinations, as well as representative mice from control groups, were used for secondary transplantation. PBMC T-cells were detected in all responder BM recipients (Fig. 3f and Table 2). To assess spleen CD3 T-cell functionality we measured proliferation in response to non-specific stimulation with concanavalin A/IL-2 or anti-CD3/CD28/IL-2. Proliferative responses were observed for both study groups (Fig. 3g and Table 2). However, as *scid* animals are well known to develop thymic lymphomas that can lead to the generation of T-cells^30^, the mere detection of T-cells in primary and secondary transplant recipients is not definite confirmation for *ex vivo* genome editing.

Conclusive evidence of low-level reconstitution with *Prkdc* gene-corrected cells was obtained by deep-sequencing in a variety of tissue samples from both primary and secondary recipients (Table 2). Two primary recipients of IPLV-ZFN + template and three of IDLV-ZFN + template showed varying levels of correction. One of the primary recipients of the IPLV-ZFN + template group had the following percentages of corrected sequences: thymus, 0.4–13.7%; blood, 5.6%; spleen, 0.45%; and spleen CD8 T-cells, 26.9%. A secondary recipient from the same group showed 5.4% in blood, 8.8% in spleen, and 24.6% in spleen CD3 T-cells. Other recipients from each ZFN + template group showed lower but detectable reconstitution levels, suggesting some stability of the gene-corrected population across the transplantation experiment.

### Off-target analyses

To study potential deleterious effects of the genome editing process we analysed off-target cutting, the presence of indels at the ZFN target site on the same haplotype as the corrected allele, and assessed the incidence of tumours. Off-target cutting was measured at 10 sites predicted by bioinformatic analysis based on data obtained by systematic evolution of ligands by exponential enrichment (SELEX; Supplementary Table 2). Samples used to evaluate off-target cutting included mock or ZFN-treated *scid* fibroblasts (with 0.05 and 18% indels at target site, respectively; Table 1), mock or ZFN-treated HSPC (0.08 and 0.60% indels at target site, respectively; Table 1), and ZFN + template treated mouse samples (Table 3), including spleen (1.23% indels, 0.07% gene correction), thymus (46.03% indels, 11.81% gene correction), and spleen CD8 T-cells (68.06% indels, 22.18% gene correction). As shown in Tables 1 and 3 for cultured cells and mice, respectively, very low levels of indels were detected at essentially all the potential off-target sites in control and ZFN-treated samples, with very small differentials between the two (differential average 0.03%, range −0.88 to 1.34%). This suggests that off-target indels detected in the ZFN-treated samples are essentially background levels due to the nature of the assays involved.

**Table 3:** On-target and off-target cutting analysis of *Prkdc* ZFN in transplanted primary mice.

Site	%Indels mouse 4.1 spleen, IDLV (background)	%Indels mouse 3.2, thymus, IPLV	%Differential (thymus-background)	%Indels mouse 3.2, spleen CD8, IPLV	%Differential (spleen CD8-background)
On-target	1.23	46.03	44.80	68.06	66.83
Off-target 1	3.92	3.04	−0.88	3.91	−0.01
Off-target 2	0.13	0.07	−0.07	0.14	0.01
Off-target 3	0.01	0.04	0.03	0.02	0.01
Off-target 4	0.05	0.05	0.00	0.04	−0.01
Off-target 5	0.12	0.05	−0.07	0.08	−0.04
Off-target 6	2.14	1.91	−0.23	1.92	−0.22
Off-target 7	0.01	0.00	−0.01	0.00	−0.01
Off-target 8	0.83	0.67	−0.16	0.74	−0.10
Off-target 9	0.05	0.39	0.34	0.04	−0.01
Off-target 10	0.95	0.76	−0.19	0.83	−0.12

Deep sequencing analysis of genomic DNA from samples treated with ZFN showed that a significant proportion of indels were a recurrent 44-bp deletion including the ZFN target site (Fig. 1a). In samples treated with ZFN + template, indels (particularly the recurrent deletion) were also observed in association to gene correction (frequencies of gene correction quoted in Table 2 exclude events associated to an indel). The percentage of gene correction with associated indel was on average 11.25% of the total gene correction frequency (range 0–100%). The recurrent deletion results in the loss of the 3′ end of exon 85, and hence presumably aberrant splicing of the final exon 86. The likely result at the protein level is an aberrant C-terminal tail downstream of the *scid* site, with unclear functional outcome. No obvious sequence microhomology is present around the deletion breakpoints which could explain the high-frequency event. One possible explanation is that, although competent for indel generation in response to ZFN-induced DSBs (as we have shown by *Cel*-I assay and deep sequencing), the defect in *Prkdc scid* cells could create a bias towards a particular microdeletion around the ZFN target site.

Potential tumorigenicity was assessed by gross pathological examination of organs after sacrifice in all animals that could be examined. We observed occasional suspected tumours in transplanted animals, including two splenomegalies in primary transplanted animals (IPLV-ZFN, IDLV-ZFN + template), and a possible leukaemia (IPLV-eGFP; control) and a thymomegaly (IDLV-ZFN + template) in secondary transplanted animals (Table 2). The latter animal was a typical case of the reported leakiness of the *Prkdc scid* model, in which T-cells can be detected in animals that have developed thymic lymphomas^30^, as we observed a thymomegaly and T-cells but failed to detect gene editing at the molecular level. We analysed by deep sequencing genomic samples of the primary transplant spleens; bone marrow, thymus and spleen from the leukaemic control animal; and thymus, spleen and PBMCs from the thymomegalic animal. In all cases we observed *Prkdc* indel frequencies at the low end of the background range, (0.00–0.04%) and no gene correction events. As (i) a suspected tumour occurred in a control animal in this experiment, (ii) a tumour in a ZFN-treated animal was a very likely spontaneous thymic lymphoma typical of this model, and (iii) there is no evidence of significant editing of the target site in any of the tumour samples from ZFN-treated animals, we conclude that the potential tumours are unrelated to ZFN or lentiviral treatment of the cells.

## Discussion

It has been recently shown that human HSPC corrected by genome editing can be transplanted into immunodeficient mice, where they regenerate an active, humanised haematopoietic system^4–7^. Demonstration of genetic modification and phenotypic rescue in animal models would be further support for such gene therapies in advance of clinical application. We have focused on a mouse model of primary immunodeficiency, with the aim of showing feasibility of genome editing as a therapeutic approach. In this *Prkdc scid* model, we have provided conclusive evidence of molecular and phenotypic correction using *scid* fibroblasts *in vitro*. We have demonstrated induction of targeted indels by the *Prkdc* ZFN through a *Cel-*I assay and deep sequencing, targeted correction in the presence of the ZFN and a corrective template by the introduction of a *Bsa*WI diagnostic restriction site and deep sequencing, as well as phenotypic rescue of DNA-PK enzymatic activity and increased resistance to melphalan DNA damage. Likewise, using HSPC in culture we have shown the introduction of targeted indels and *Prkdc* gene correction.

We have transplanted the gene-edited HSPC into irradiated *Prkdc scid* recipients to analyse the efficiency of *ex vivo* rescue. Some primary recipients of the ZFN + template groups have shown increasing levels of CD3, CD4 and CD8 T-cells. At culling, some of the mice also showed increased levels of double-positive CD4/CD8 T-cells in the thymus. Some of the secondary transplant recipients have also shown enhanced levels of CD3, CD4 and CD8 cells. While these are important observations regarding possible rescue of T-cells, *Prkdc scid* animals are well known to develop thymic lymphomas that can lead to the generation of T-cells^30^ and hence the presence of T-cells *per se* is not conclusive proof of rescue. These immunodeficient animals also lack B-cells, but we have not observed recovery of B-cell levels. This was not entirely unexpected, as observations in immunodeficient mice indicate that significantly higher levels of gene addition in HSPC are required to rescue B-cells compared to T-cells^31^. A similarly favoured rescue of T- over B-cell deficiency has been observed in some clinical trials of gene addition therapy, albeit this may be at least in part related to absence of conditioning^32^.

Definite proof of the ability of gene-edited HSPC to mediate some level of reconstitution upon transplantation has been obtained by deep sequencing of animal tissues from some of the recipients. The expected signature for genome editing (correction of the *scid* mutation associated to the introduction of the *Bsa*WI diagnostic site) has been observed in blood PBMC, thymus, spleen and purified spleen CD3 or CD8 T-cells. Levels are variable in the positive primary transplanted animals, higher with IPLV-ZFN and were also observed in secondary recipients. We consider the presence of the genome editing signature proof that reconstitution can be achieved, albeit inefficiently both in terms of the number of positive animals and the levels in different tissues.

Pharmacological inhibition of DNA-PKcs has been shown to increase the frequency of CRISPR/Cas9-mediated genome editing^33^; thus, it is possible that in *Prkdc*-deficient cells the defect in NHEJ favours HDR genome editing. It is also conceivable that *Prkdc* deficiency could contribute to the recurrence of the 44-bp deletion including the ZFN target site that we have observed in multiple samples from genome editing *in vitro* and *ex vivo*. Inhibition of DNA-PK during repair of CRISPR/Cas DSBs has been reported to inhibit canonical NHEJ and promote alternative (microhomology-mediated) NHEJ, resulting in a bias towards larger deletions^34^. These results highlight the importance of considering the possible effect of DNA damage repair defects (and targeted manipulation of the DSB repair systems^34^) on genome editing strategies. We have alleviated concerns over cytotoxicity and genotoxicity by showing background levels of off-target indels in *scid* fibroblasts and HSPC treated with the ZFN. We have also analysed the expected, occasional tumours observed in the irradiated, transplanted immunodeficient animals, showing they appear unrelated to ZFN treatment.

We have shown that some level of reconstitution can be achieved using *ex vivo* genome editing with ZFN and *Prkdc* template delivered with IDLV, an advantageous non-integrating system which will be diluted out as the gene-edited population expands. Reconstitution was also achieved with IPLV-delivered ZFN, somewhat surprisingly. Reconstitution has not been achieved in all primary recipients of gene-edited cells, so significant improvements in efficiency are required. In conclusion, a mouse model of primary immunodeficiency can be partially reconstituted by *ex vivo* HSPC genome editing and transplantation. The tremendous expansion in the genome editing field witnessed over the last few years^35,36^ attests to the power of the technology and forecasts fast advance of ground-breaking HSPC gene repair towards the clinic, where ZFN-mediated gene disruption in T-cells is already showing excellent results in the context of therapy for HIV^37^.

## Methods

### ZFN design and production

ZFNs targeting *Prkdc* exon 85 were designed by modular assembly using an archive of zinc finger proteins. Optimised zinc-finger domains were linked to obligatory heterodimeric *Fok*I domains^38^. ZFN genes were cloned and initially tested on pVAX plasmid backbones, and then transferred onto self-inactivating lentiviral transfer plasmids pCCLsc_S_W (where transgene is driven by SFFV promoter and followed by Woodchuck hepatitis virus post-transcriptional regulatory element, WPRE) or pRRLsc_C_W (CMV promoter and WPRE).

### Targeting constructs

Two *Prkdc* repair templates were made: (i) a classical, pBlueScript plasmid-based long construct including a *neo* gene within the homology region, and (ii) a short, lentiviral vector-based PCR-amplified repair template with a diagnostic restriction site. The long construct included the 7.5-kb *Hin*dIII fragment spanning *Prkdc* exons 82–86, subcloned from a wild-type yeast artificial chromosome (YAC) previously shown to complement the *scid* mutation in cell culture^39^. A *neo* cassette expressed from a PGK promoter was inserted in intron 85, at a *Pml*I site, allowing for positive selection after calcium phosphate-mediated plasmid transfection. The short *Prkdc* template was constructed by PCR amplification of a 1,626 bp sequence from wild-type Balb/c mouse fibroblast genomic DNA using forward and reverse primers 5′-AATGTTTAGTTTTTATGAGTGTTTTGC-3′ and 5′-CAAGCCATCTCTCTAGCCCTAC-3′, respectively. Two silent point mutations were introduced via site-directed mutagenesis 3- and 6-bp upstream of the *scid* site to create a diagnostic *Bsa*WI restriction site (Site-Directed Mutagenesis Kit, Stratagene, USA). The short *Prkdc* template was cloned into the self-inactivating lentiviral vector plasmids, which then encoded either the *Prkdc* template alone or one ZFN monomer and the *Prkdc* template together. The template was cloned upstream of the central polypurine tract/central termination sequence (cPPT/cTS) and in reverse orientation.

### Cells, transfection and transduction

HEK293T, HeLa, wild-type and *scid* mouse fibroblasts were maintained in DMEM with stable glutamine and 4.5 g/l glucose supplemented with 10% FBS and 1% penicillin/streptomycin at 37 °C and 5% CO_2_. *Tert*-immortalised *scid* fibroblasts, obtained by transduction with a retroviral vector encoding mouse *Tert* and puromycin-acetyltransferase (*pac*) were maintained likewise but in the presence of 3 μg/ml puromycin and used in all fibroblast experiments. Wild-type and *scid* HSPC were obtained from bone marrow, by flushing the femur and tibia of 6 to 8-week-old Balb/cOlaHsd or Balb/cJHan(tm)Hsd-*Prkdc scid* mice. Lin^−^ HSPC were isolated using a MACS Lineage cell depletion kit (Miltenyi Biotec, Germany) as directed by the manufacturer. HSPC were cultured without splitting, in 24-well non-tissue culture-treated plates at 37 °C and 5% CO_2_, using serum-free StemSpan medium (Stemcell Technologies) containing 100 ng/ml murine stem cell factor (SCF), 100 ng/ml murine Fms-related tyrosine kinase 3 (Flt-3), 20 ng/ml human interleukin-6 (IL-6) and 1% penicillin/streptomycin. Fibroblast transfections for genome editing experiments were done using 10^6^ cells/plate (*n* = 3), calcium phosphate co-precipitation and 10 μg (template plasmid in transfection/transduction experiments) or 30 μg of DNA (10 μg of each if co-transfecting ZFN monomer and template plasmids, using an irrelevant plasmid to normalise total amount of DNA in controls). Cells were transduced with IPLV or IDLV in the presence (HeLa, fibroblasts) or absence (HSPC) of 8 μg/ml polybrene, and then processed for analysis or injection as indicated. Fibroblast genome editing experiments involving ZFN vector transduction and template plasmid transfection were done with an optimum 48 h delay before the latter was carried out. 800 μg/ml G418 added 48 h post-transfection was used for selection, and colonies were either stained with crystal violet for counting 9 d post-transfection or picked and expanded for PCR analysis of *Prkdc* gene editing.

### Lentiviral vector production

Third-generation lentiviral vectors were prepared as described^40,41^. Briefly, HEK293T cells were co-transfected with four plasmids by calcium phosphate precipitation, at a molar ratio of 1:1:1:2 (packaging:rev:envelope:transfer). For IPLVs the packaging plasmid was pMDLg/pRRE and for IDLVs it was pMDLg/pRRE-intD64V^41^. Rev was delivered on pRSV-rev and the vectors were pseudotyped with VSV-G protein using plasmid pMD2.VSV-G. The transfer plasmids encoded either the short *Prkdc* template alone, one of two ZFN monomers or the template and one of the monomers. Vector particles were concentrated by ultracentrifugation and titrated by real time-PCR using HeLa cell transductions^41^.

### Western blot

Cells were homogenized in RIPA lysis buffer containing protease and phosphatase inhibitors. Protein concentrations were determined using the DC protein assay (Bio-Rad) and proteins ran on 5–12% acrylamide gradient gels. For FLAG epitope detection, the transferred membrane was incubated with mouse anti-FLAG M2 monoclonal antibody (Stratagene, UK) at 1:1000 dilution, using IRDye® 800CW goat anti-mouse (Li-cor GmbH, Germany) at 1:2000 dilution as secondary antibody. For loading controls, rabbit polyclonal anti α-tubulin (Abcam, UK) at 1:2000 dilution was used as primary antibody, and then Alexa Fluor® 680 Goat anti-rabbit (Invitrogen, USA) at 1:5000 dilution as secondary antibody. Blots were scanned at 700 and 800 nm channels for α-tubulin and anti-FLAG, respectively, using an Odyssey imager (Li-cor Bioscience, Germany).

### *Cel*-I assay

ZFN-induced indels were detected using the SURVEYOR mutation detection kit (Transgenomic, USA) as described previously^42^. At day 3 post-transduction, genomic DNA from 1 × 10^6^ mock- or ZFN-treated *scid* fibroblasts or HSPC was extracted using DNeasy kit (Qiagen, Germany) and PCR-amplified using forward and reverse primers 5′-GCAGACAATGCTGAGAAAAGG-3′ and 5′-GCACAAAACAGACAAGGGTGT-3′, respectively. Thermocycling conditions were as follows: 95 °C, 5 min; 95 °C, 30 s, 60 °C, 30 s, and 68 °C, 40 s for 35 cycles; and a final extension step of 68 °C for 2 min. The PCR product was denatured and allowed to reanneal, and processed as per instructions of Surveyor kit. The final products were analysed by polyacrylamide gel electrophoresis to assess ZFN-induced indels, using Image J/V2 software to quantify the intensity of the bands.

### PCR screening for plasmid-based *Prkdc* gene editing

Following plasmid transfection or lentiviral transduction/plasmid transfection of ZFN monomer genes and the long *Prkdc-neo* targeting template, G418-resistant colonies were screened using an inside-out PCR strategy. Genomic DNA was extracted with the DNeasy^®^ kit (Qiagen). The forward primer (5′-TCGCCTTCTTGACGAGTTCT-3′) hybridised within the *neo* cassette, whereas the reverse primer (5′-TTTTCCCCCTCATGTCACTC-3′) was directed to *Prkdc* genomic DNA downstream of the template right homology arm. The amplicon length was 1335 bp. For PCR amplification, 50 µl reactions were prepared containing 10 µl 5X GoTaq^®^ (Promega, UK) reaction buffer, 1.5 µl of 10 mM dNTPs, 4 µl of 25 mM MgCl_2,_ 1.5 µl of 10 µM of each forward and reverse primer, 200 ng DNA template, and 0.5 µl of 5 u/µl GoTaq DNA polymerase. Thermo-cycling conditions were: initial denaturation at 95 °C for 2 min, then 35 cycles of 95 °C for 45 s, 59 °C for 60 s, 72 °C for 30 s, followed by final extension at 72 °C for 5 min. PCR products were purified using a PCR purification kit (Qiagen) and digested with restriction enzymes predicted to cut the amplicons.

### *Bsa*WI assay

*Prkdc* gene editing was measured by gel assay as described previously^26^. Briefly, genomic DNA from mock- or ZFN/template-treated *scid* fibroblasts or HSPC was extracted using DNeasy kit (Qiagen, Germany), and 100 ng subjected to PCR using primers external to the *Prkdc* template (forward 5′-AACAATCCTCCTCCGAACCT-3′ and reverse 5′-TGGAGGTGGAAGAACCAAAC-3′). Thermocycling conditions were as follows: 95 °C, 30 s; 95 °C, 30 s, 59 °C, 60 s, and 65 °C, 75 s for 35 cycles; and a final extension step of 65 °C for 10 min. The PCR products were digested overnight with *Bsa*WI (NEB, UK) and separated on a 0.7% agarose gel. The DNA was transferred overnight onto GeneScreen Plus Membrane (Perkin Elmer, USA), hybridised overnight using as probe [α-32P]-ATP labeled *Prkdc* template, washed and exposed to a phosphoimager screen. The screen was scanned using a Typhon-8600 (Amersham Pharmacia Biotech, UK) and the bands were quantified using Image J/V2 software^26^.

### DNA-PKcs Assay

The SignaTECT DNA-dependent protein kinase assay system (Promega, USA) was used as previously described^27^. Nuclear proteins from mock- or ZFN/template-transduced *scid* fibroblasts were extracted using the CelLytic Nuclear Extraction kit (Sigma Aldrich, USA), and processed as per SignaTECT system instructions. Biotinylated peptide substrates were captured by spotting reactions onto individual squares of high binding capacity capture membrane. After several washes of the membrane, DNA-PK activity was quantified using the above phosphoimaging system.

### MTT assay

Cellular viability was determined by the MTT assay as described previously^43^. Wild-type and *scid* fibroblasts were exposed to increasing concentrations of melphalan for 1 h, washed, and then cultured for a further 5 days. Cells were then exposed to the MTT tetrazolium salt for 4 h at 37 °C, and the formation of formazan was measured at 560 nm using a microplate reader (Promega, USA). The concentration giving the maximum difference between wild-type and *scid* fibroblast viability was chosen for selection. All values are averages of 3 independent experiments each done in duplicate.

### Colony Forming Unit (CFU) assay

10^5^ mock- or ZFN/template-transduced *scid* fibroblasts were cultured in the presence of 10 μM melphalan (optimal dose determined using MTT assay) for 1 h, and the treatment was repeated once a week for three weeks, until colonies formed. Colonies were either stained using crystal violet and counted or pooled for analysis using the gene editing *Bsa*WI assay; *n* = 3.

### Deep sequencing analysis

Deep sequencing was performed using the MiSeq system (Illumina, San Diego CA). Specific primer pairs (Supplementary Table 3) were designed, each amplifying 100–200 bp fragments spanning the target site or one of the predicted top 10 putative off-target sites (Supplementary Table 3), and each incorporating adaptor sequences for the MiSeq processing. Briefly, genomic DNA samples were initially amplified using primers listed in Supplementary Table 3, then a nested adaptor PCR was run using the primers listed in the footnote to Supplementary Table 3. Following amplifications, PCR products were barcoded by running a 14-cycle barcode PCR. The barcoded PCR products were purified using a PCR purification kit (Qiagen), pooled and sequenced using the Illumina MiSeq platform. A custom-written computer script was used to extract “high-quality sequence reads”, based on their alignment with the wild-type template sequence. Sequences that contained a deletion or insertion resulting in shorter or longer amplicons which involve any base in the ZFN cleavage site were classified as an NHEJ-mediated deletion or insertion, respectively; together, such mutations are classified as “indels”. Sequences with the expected changes (3 substitutions, 1 to wild-type and 2 for novel *Bsa*WI site (Fig. 1a) were classified as “corrected”.

### HSPC transplantation

All experiment procedures were approved by the Institutional Research Ethics Committee (Institute of Child Health, University College London, UK) and performed according to UK Home Office Animal Welfare Legislation. For primary transplantation, cells were collected 16 h post-transduction, washed twice in phosphate-buffered saline (PBS) and resuspended in PBS. Female BALB/cJHan(tm)Hsd-*Prkdc scid* mice were sub-lethally irradiated (2.5 Gy) and then transplanted with ~1 × 10^6^ male cells in 200 µl PBS by tail vein injection (*n* = 5 per transplant group; Supplementary Table 1). For secondary transplantation, 32 weeks post-primary transplantation, whole bone marrow of control and responder mice was isolated and ~1 × 10^6^ cells transplanted by tail vein injection onto female Balb/cJHan(tm)Hsd-*Prkdc scid* mice previously sub-lethally irradiated (2.5 Gy; *n* = 2 for wild-type transplant, *n* = 3 for IPLV-eGFP transplant, *n* = 4 for IPLV-ZFN and IDLV-ZFN groups). Blood samples were collected at different intervals by tail vein withdrawal. Table 2 includes animals for which molecular data are available.

### Engraftment and vector copy number measurements

Engraftment of male donor-derived cells in the female recipient mice was calculated against standard curve with known percentage of male DNA isolated from C57 Balb/c male mice bone marrow. DNA was extracted using the Qiagen DNeasy® kit. A 102-bp sex-determining region (*Sry*) on the Y chromosome was amplified by qPCR using primers forward 5′-TCATCGGAGGGCTAAAGTGTCAC-3′ and reverse 5′-TGGCATGTGGGTTCCTGTCC-3′. Thermocycling conditions were: 95 °C for 10 min, 40 cycles of 95 °C for 15 s, 60 °C for 20 s, 72 °C for 20 s. A melting curve analysis was performed at the end with continuous reading between 50 and 100 °C. *Titin* was used as a reference gene to normalise the total number of cells. qPCR was carried using primers forward 5′-AAAACGAGCAGTGACGTGAGC-3′ and reverse 5′-TTCAGTCATGCTGCTAGCGC-3′. Cycling conditions were: 95 °C for 10 min, 40 cycles of 95 °C for 15 s, 60 °C for 60 s, 72 °C for 15 s, followed by a melting curve analysis.

To analyze the number of vector integration events (vector copy number), DNA was prepared using the DNeasy kit (Qiagen) according to the manufacturer’s instructions. The vector-specific WPRE was amplified with forward primer 5′-TGGATTCTGCGCGGGA-3′, and reverse 5′-GAAGGAAGGTCCGCTGGATT-3′ with the probe 5′-FAM-CTTCTGCTACGTCCCTTCGGCCCT-TAMRA-3′ (Eurofins). The WPRE copy number was normalized against the PCR product of mouse *titin* exon 5, which was amplified with the primers described above with the addition of the probe 5′-FAM-TGCACGGAAGCGTCTCGTCTCAGTC-TAMRA-3′ (Eurofins) for quantification. The real-time PCR was performed on samples and plasmid standards which contained both target genes, using 2 µl DNA, 1x Taqman Universal Mastermix (Applied Biosystems), 900 nM each of forward and reverse primers and 200 nM FAM-labeled probe in a 20 μl reaction (in triplicate) and analysed using the ABI Prism 7000 Real Time PCR System (Applied Biosystems, California, USA).

### Flow cytometry analysis

Peripheral blood mononuclear cells were labelled with anti-mouse CD3-PECy7 (or Pacific blue) antibody, anti-mouse CD4-PE or anti-mouse CD8-APC. Flow cytometry data were acquired with a CyAn ADP (Beckman Coulter, USA) and analyzed using FlowJo software (TreeStar Inc, Ashland, OR, USA).

### T-cell proliferation assay by CFSE dilution analysis

CD3 T-cell splenocytes were purified by magnetic capture and incubated in RPMI-1640 (Sigma) supplemented with 100 IU/ml penicillin, 100 μg/ml streptomycin, 2 mM L-glutamine, 0.01 M Hepes, 50 μM 2-β-mercaptoethanol (Gibco-Invitrogen), 10% heat-inactivated FCS (EuroClone, UK), supplemented with 1 μM CFSE (Celltrace, Invitrogen). The efficiency of CFSE staining was greater than 99%. CFSE-labelled cells were then incubated in the presence of 3 µl/ml ConA (Sigma) and 100 IU/ml IL-2 (R&D Systems) or 0.5 µl mouse CD3/CD28 T Expander (Dynal Biotech, Invitrogen) and 100 IU/ml IL-2. After 72 h, expanded cells were labelled with anti-CD3-PE (145–2C11 clone; BD Pharmingen). CFSE dilution in the T-cell population was measured by flow cytometry using an LSR Fortessa cell analyzer (Becton Dickinson) running FACSDiva software, and data processed using FlowJo software (TreeStar Inc, Ashland, OR, USA).

### Data availability

The datasets generated during the current study are included in this published article (and its Supplementary Information files) or available from the corresponding author on reasonable request.

## Electronic supplementary material

Supplementary information
