# Supplementary material for: Molecular Evidence of Genome Editing in a Mouse Model of Immunodeficiency
Source: Sci Rep. 2018 May 29;8:8214. doi: 10.1038/s41598-018-26439-9 (PMC5974076; doi:10.1038/s41598-018-26439-9)
Supplement: Supplementary file 1 — Supplementary information [file 41598_2018_26439_MOESM1_ESM.pdf]

Molecular Evidence of Genome Editing in a Mouse Model of Immunodeficiency

H.H. Abdul-Razak[1+], C.J. Rocca[1+], S.J. Howe[2,3], M.E. Alonso-Ferrero[2], J. Wang[4], R. Gabriel[5], C.C. Bartholomae[5], C.H.V. Gan[2], M.I. Garín[6], A. Roberts[7], M.P. Blundell[2], V. Prakash[1], F.J. Molina-Estevez[1,6], J. Pantoglou[1], G. Guenechea[6], M.C. Holmes[4], P.D. Gregory[4], C. Kinnon[2], C. von Kalle[5], M. Schmidt[5], J.A. Bueren[6], A.J. Thrasher[2,8] and R.J Yáñez-Muñoz[1,*]

[1]AGCTlab.org, Centre for Biomedical Sciences, School of Biological Sciences, Royal Holloway, University of London, Egham, UK.

[2]Infection, Immunity, Inflammation and Physiological Medicine Programme, Molecular and Cellular Immunology Section, UCL Great Ormond Street Institute of Child Health, University College London, London, UK.

[3]Gene Transfer Technology Group, UCL Institute for Women's Health, University College London, London, UK.

[4]Sangamo Therapeutics, Inc., Richmond, California, USA.

[5]Department of Translational Oncology, National Center for Tumor Diseases and German Cancer Research Center, Heidelberg, Germany.

[6]Division of Hematopoietic Innovative Therapies, Centro de Investigaciones Energéticas, Medioambientales y Tecnológicas (CIEMAT)/Centro de Investigación Biomédica en Red de Enfermedades Raras (CIBERER-ISCIII)/Instituto de Investigación Sanitaria Fundación Jiménez Díaz (IIS-FJD, UAM), Madrid. Spain.

[7]Department of Medical and Molecular Genetics, King's College London, London, UK.

[8]Great Ormond Street Hospital NHS Foundation Trust, London, UK.

*rafael.yanez@royalholloway.ac.uk

[+]these authors contributed equally to this work

SUPPLEMENTARY INFORMATION

Supplementary Table 1 | Animal groups used in transplantation experiment.

Group #	Group name	Donor cells (male)	MOI	Recipients (female)
1	wt control	BALB/c OlaHsd	-	BALB/c JHan(tm)Hsd-*Prkdc scid*
2	e*GFP* control	BALB/c JHan(tm)Hsd-*Prkdc scid* transduced with CMV-eGFP-WPRE IPLV	400	BALB/c JHan(tm)Hsd-*Prkdc scid*
3	IPLV	BALB/c JHan(tm)Hsd-*Prkdc scid* transduced with IPLV ZFN monomers and IDLV template.	100: 100: 200	BALB/c JHan (tm)Hsd-*Prkdc scid*
4	IDLV	BALB/c JHan(tm)Hsd-*Prkdc scid* transduced with IDLVs template/CMV ZFN1 and template/CMV ZFN2	500: 500	BALB/c JHan(tm)Hsd-*Prkdc scid*

Supplementary Table 2 | SELEX amplicons for off-target analysis

Rank	Name	Score	Chromosome	Location	Site	Mismatch (bp)	Arrangement (Left ZFN / Gap / Right ZFN)	Left_Primer	Right_Primer	Amplicon Length (bp)
1	On-target	8.22E-11	chr16	15839293	GGGCCAaCCCaGCTGTTATAACtTGGtaAGACTTGT	5	17834 / 6 / 17373_1bpSKIP	TGAGCAGACAATGCTGAGAAA	AACAGACAAGGGTGTGAGCC	301
2	Off-target 1	1.33E-11	chr4	136870923	CtcCCtgCCCCGCCAAGCCcCCAGGATGGACTTGG	5	17834 / 5 / 17373_1bpSKIP	ACTTCACCAAATCACCCAGC	ATCTCAGCCATTCAACACCC	303
3	Off-target 2	8.03E-12	chr15	91433193	CCAAGTCCATCCAGGTTTTAGGaCtGtGGTGGggG	5	17373_1bpSKIP / 5 / 17834	GGGAAGGAAAGGCAATCTCT	GCTGACTATGAGGAGCGAGG	387
4	Off-target 3	6.01E-12	chr7	117849399	CaGtGTCTATCCAaGTTGCCTTGgaGAGGTGGCCT	5	17373_1bpSKIP / 5 / 17834	CCAAACTGGAGAATGGCTGT	GAGCACTAAGCTGGGGGAG	327
5	Off-target 4	4.82E-12	chr7	90098094	GCAAGTCTAaCCAGGTGTGGCTGCGGGaaTGGCtC	4	17373_1bpSKIP / 5 / 17834	TTGTTCTGACGATGCTCCTG	AGCTCGGAGACAAGGAAACA	341
6	Off-target 5	4.35E-12	chr6	85095172	ACAGGaCAgTCCAGGTGGGAGAGCtGGGGaGGCtA	5	17373_1bpSKIP / 5 / 17834	CAGCTTTAGGGCACTTTTGC	TCTTCACACCTTCCCTGCTT	314
7	Off-target 6	2.45E-12	chr15	98526028	CgGAGTCTATCgTGGTGGCGGCGGCGGcGGTGGCgG	4	17373_1bpSKIP / 6 / 17834	CTGGACACAGACCCTGGATT	GTAACCCTGGCTGTTCTGGA	300
8	Off-target 7	2.07E-12	chr1	91082864	GaGCCACCTCtGaCTGCTCACCAGGATAGgCCTGT	4	17834 / 5 / 17373_1bpSKIP	CGATGGCTGAATGTATGCAC	TGTGCTATATAGGTGGGGGC	300
9	Off-target 8	2.02E-12	chr13	45481996	CaGCCtCCTCCtCCACGCGCACCAGGAgAaACCTGG	5	17834 / 6 / 17373_1bpSKIP	CGGTGGGTTTTCTATTCCCT	CTTCACAAAGCAGAAGCGTG	360
10	Off-target 9	1.84E-12	chr6	39217052	GtGCCAaCCCaagTTCTATCACCTGGAcTGACaTGG	7	17834 / 6 / 17373_1bpSKIP	AGGCCTGCATCTGTATGACC	ACAAAGGTATGCCAGACAGGA	383
11	Off-target 10	1.74E-12	chr8	11441154	AGGCCACCCCCcCCCCAACAcCaTGGATAGAgCTGA	4	17834 / 6 / 17373_1bpSKIP	CTACCATGCTCTCTGTGGCA	GGCTACTTACCTGGGGTTCC	335

Supplementary Table 3 | MiSeq adaptor PCR primers

Target	Chrom	Location	Forward Primer‡	Reverse Primer#
On-Target	chr16	15839293	CGGAAAAGAATTGGTATCCAC	CTGCTCAGAAGTGTGTGAAGTGC
Off-target 1	chr4	136870923	GCTTCAGTCATTACACGCCC	CTCAGCCATTCAACACCCC
Off-target 2	chr15	91433193	GGAGAGGAAGTCTTCCACGG	GAAACCTTCTGTGGCAACCC
Off-target 3	chr7	117849399	CCTGTCAGGTCTGGAGGGTA	AAGGTTCTTGAATGAAGTTGGG
Off-target 4	chr7	90098094	GCTGCACTGATGGGTCTGGT	GTTCATGCTTGGCTCATTCC
Off-target 5	chr6	85095172	CCCTTCCTGCCTGGGATTT	GCTAAAGGAGGAGGAGGAGGAG
Off-target 6	chr15	98526028	GCTACCAGAACAATGTCCCTG	CTCAACCTGGCAGAGATCCAC
Off-target 7	chr1	91082864	GAGACCTCAGTCACGGTTCATT	GACACTTGCTGTAGACAAAGAAGG
Off-target 8	chr13	45481996	GGAAGAAATGACAGGAGGGAAG	GTTAAAAGCAGAAGGCCAGG
Off-target 9	chr6	39217052	CCCAGAATTCCACATACAAAACA	GCATGAGGAGGTCAGAGGTC
Off-target 10	chr8	11441154	GCGACTGCCTCAGTTTCTCTAC	GGTCTCCATGAGCATCAACACC

‡ Forward primer sequence: 5'- CTTTCCCTACACGACGCTCTTCCGATCTnnnnn – followed by target-specific sequences as listed.

# Reverse primer sequence: 5'- GACGTGTGCTCTTCCGATCT – followed by target-specific sequences as listed.

Supplementary Figure 1

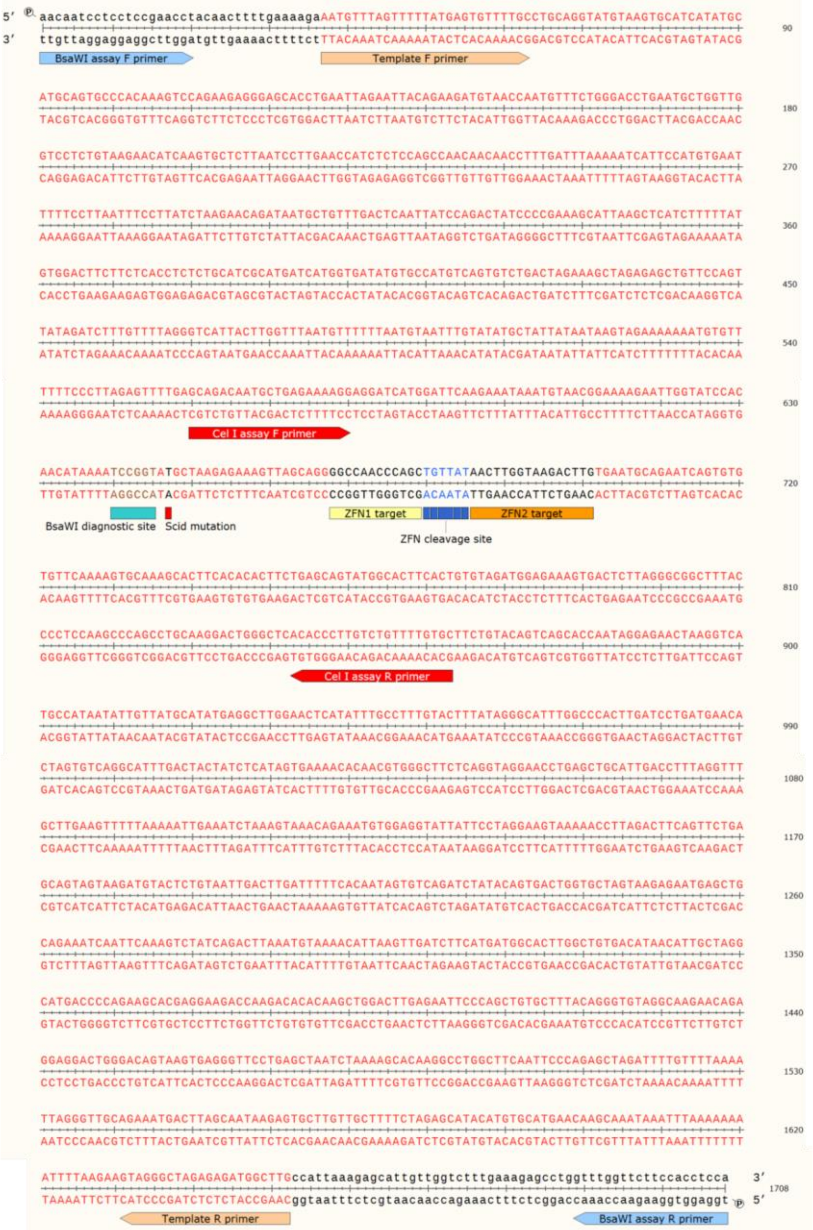

Supplementary Figure 1 | Genomic *Prkdc* sequence flanking *scid* site after genome editing. Indicated are the gene editing template (red sequence), the *scid* site showing the wild-type sequence, the location where the engineered diagnostic *Bsa*WI site was introduced, the binding sites for the ZFN monomers and predicted cut site, as well as forward (F) and reverse (R) PCR primers for *Bsa*WI assay (also used for deep sequencing), amplification of the 1.6 kb targeting template and indel *Cel*-I assay.

Supplementary Figure 2

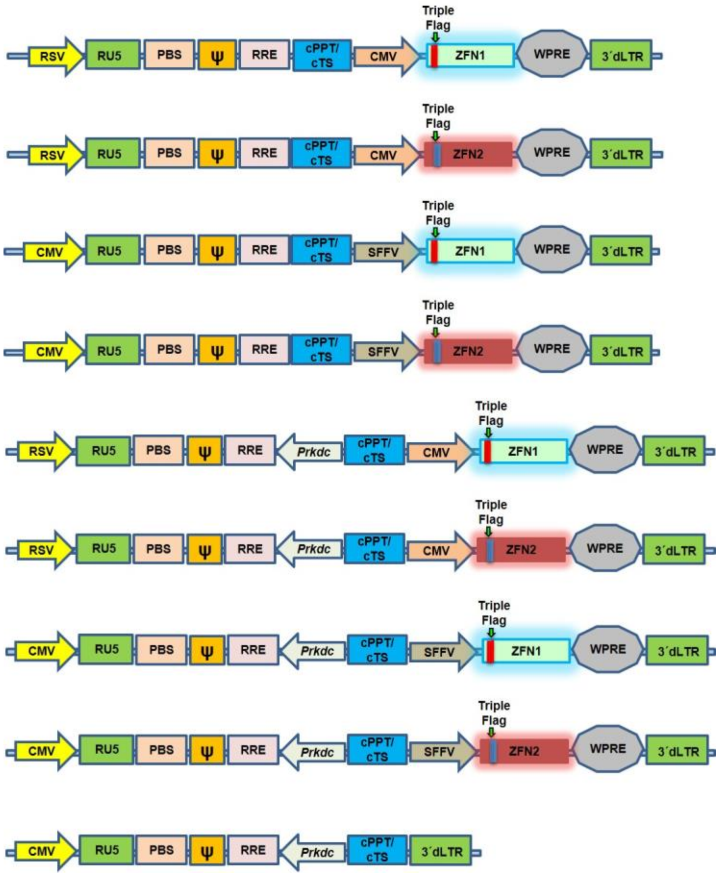

Supplementary Figure 2 | Schematic of lentiviral plasmid constructs. Plasmid backbones are not shown. Abbreviations: RSV, Rous sarcoma virus promoter; CMV, immediate early cytomegalovirus promoter; SFFV, Spleen focus-forming virus LTR promoter; RU5, 3'end of HIV long terminal repeat (LTR), including repeat (R) and unique 5 (U5) regions; PBS, primer binding site; RRE, rev response element; ψ, encapsidation signal; cPPT/cTS, central polypurine tract/central termination sequence; ZFN1 and ZFN2, Zinc-Finger Nuclease monomer open reading frames including N-terminal triple FLAG epitopes; WPRE, Woodchuck hepatitis virus post-transcriptional regulatory element; 3'dLTR, HIV LTR with self-inactivating (SIN) internal deletion within unique 3 (U3) region that essentially eliminates promoter activity, making gene expression in the resulting provirus dependent on an internal promoter; *Prkdc*, repair template, cloned in reverse orientation to prevent splicing of exon 85 during the lentiviral vector RNA stage.

Supplementary Figure 3

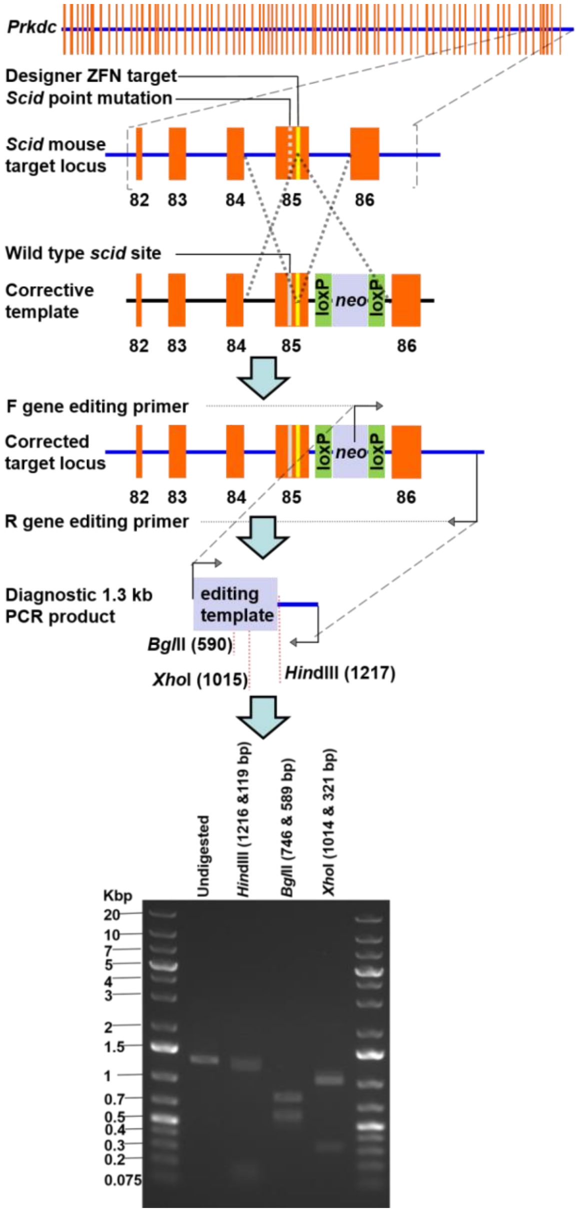

Supplementary Figure 3 | Schematic of *neo Prkdc* gene editing PCR assay. Following gene editing with the plasmid-based *neo*-containing template, genomic DNA was extracted and PCR amplified using a forward primer internal to *neo* and a reverse primer downstream from 3' homology arm. PCR products were separated by gel electrophoresis, before or after digestion with suitable enzymes. A 1.3 kb, digested by *Hin*dIII, *Bgl*II and *Xho*I, is diagnostic for gene targeting.

Supplementary Figure 4

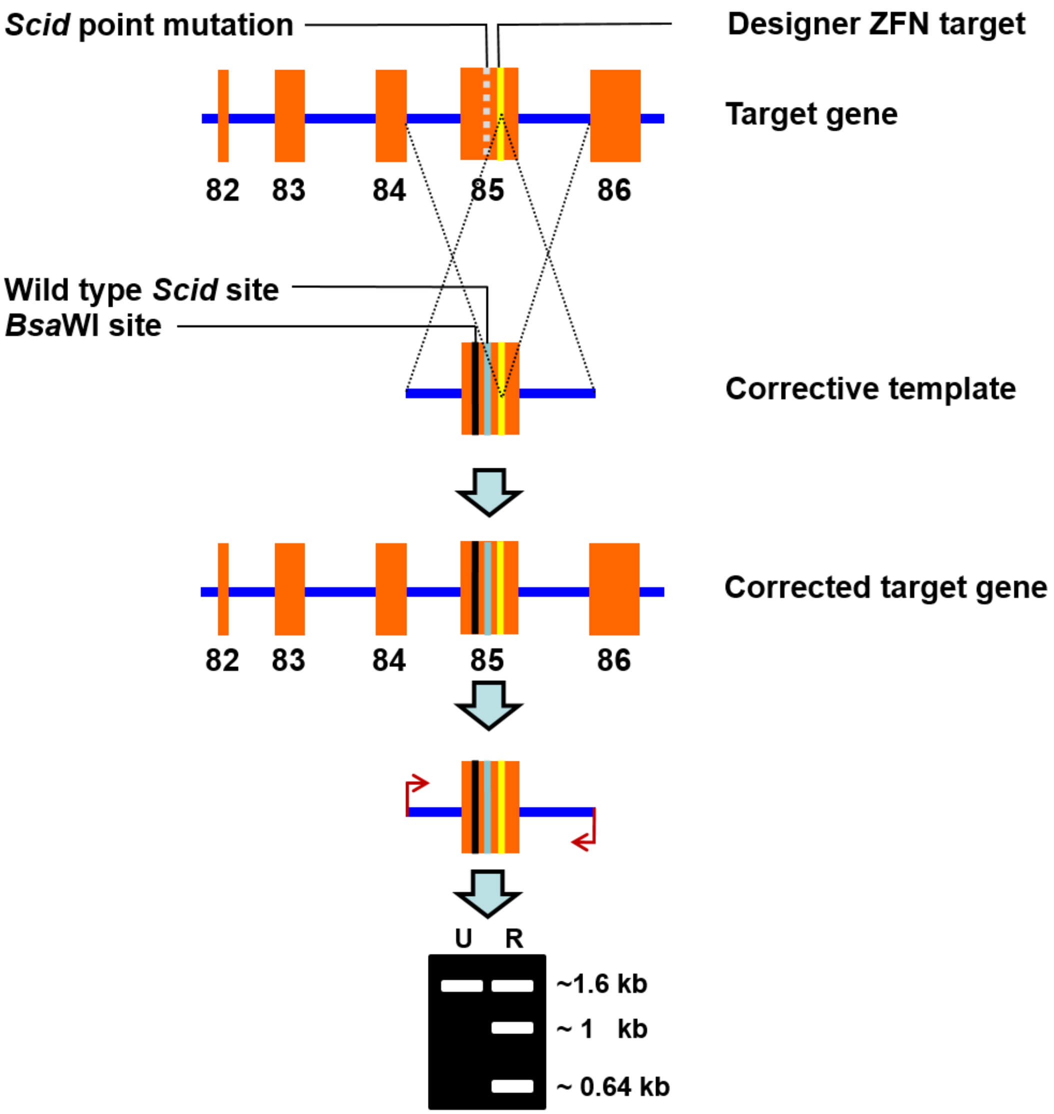

Supplementary Figure 4 | Schematic of *Prkdc* gene editing *Bsa*WI assay. Following gene editing, genomic DNA was extracted and PCR amplified using primers shown on Supplementary, Figure 1. Amplicons were digested with *Bsa*WI, separated by gel electrophoresis, blotted, transferred onto nylon membrane, hybridised with radio-labelled probe (the original PCR product) and imaged. U: Unrepaired, R: Repaired. The presence of 1 and 0.64 kbp bands is diagnostic for gene editing; in practice the 0.64 kbp band is difficult to visualise against low molecular weight smear so we only rely on 1 kb band for quantification of gene editing frequency.

Supplementary Figure 5

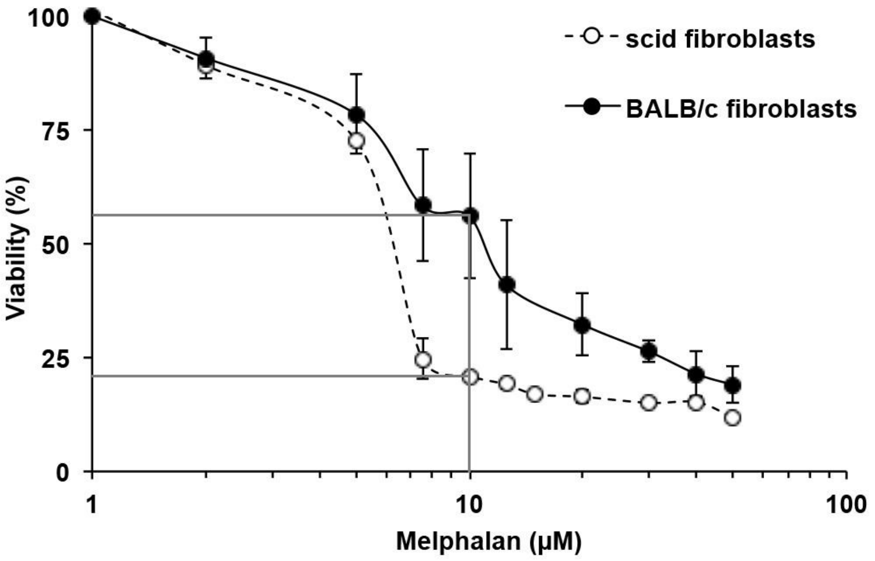

Supplementary Figure 5 | Viability of balb/c and *scid* fibroblasts after melphalan treatment. *mTert scid* and balb/c fibroblasts were exposed to melphalan for 1 h and cultured for 5 further days in drug-free medium, before an MTT assay to determine cell viability.

Supplementary Figure 6

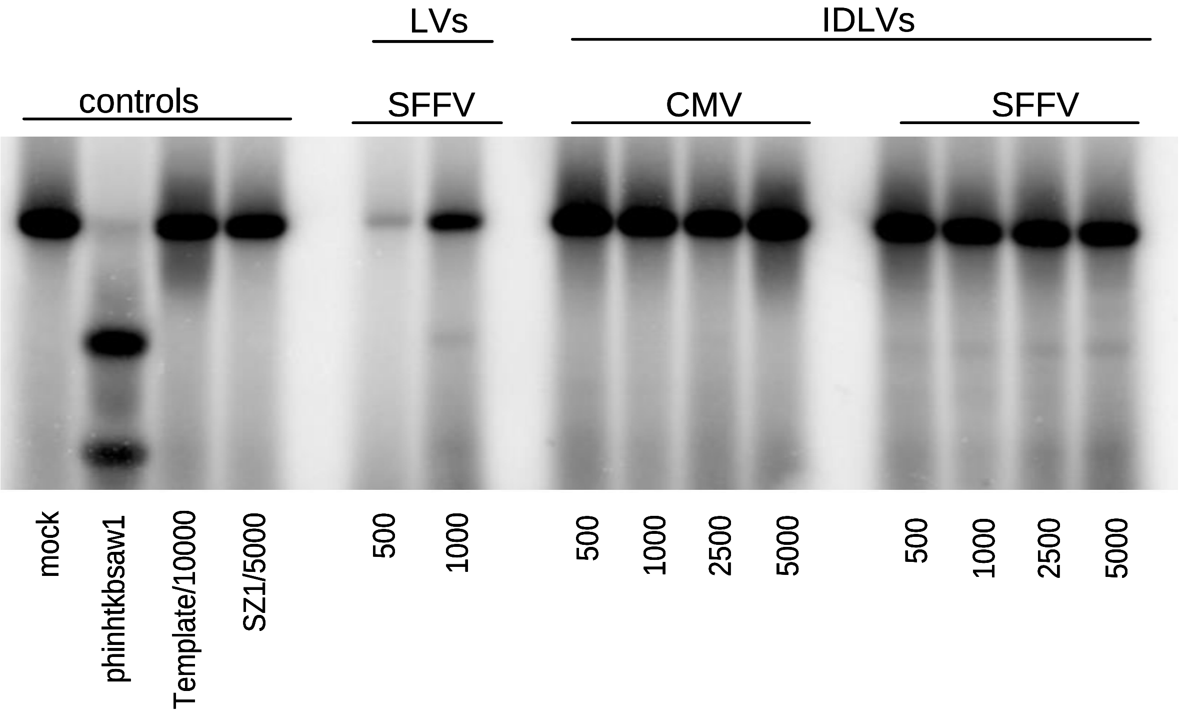

Supplementary Figure 6 | *Prkdc* gene editing in *scid* fibroblasts. The uncropped gel from Figure 2b is shown. Cells were transduced with IPLV-ZFN/IDLV-template or IDLV-ZFN/template at the indicated MOI and with ZFN genes driven by the indicated promoters, and genomic DNA was extracted 10 d post-transduction. *Scid* locus was PCR-amplified with primers external to template, and ZFN-mediated gene correction was quantified from the diagnostic *Bsa*WI band (arrow) and shown as %*Prkdc* correction. LV denotes standard integration-proficient lentiviral vector (IPLV).
